# Effects of nitrogen and phosphorus addition on growth and leaf nitrogen metabolism of alfalfa in alkaline soil in Yinchuan Plain of Hetao Basin

**DOI:** 10.7717/peerj.13261

**Published:** 2022-04-13

**Authors:** Gu Xudong, Zhang Fengju, Wang Teng, Xie Xiaowei, Jia Xiaohui, Xu Xing

**Affiliations:** 1 School of Agriculture, Ningxia University, Yinchuan, China; 2 School of Ecology and Environment, Ningxia University, Yinchuan, China

**Keywords:** Alfalfa, Nutrient, Nitrogen metabolism, Alkaline soil, Fertilization

## Abstract

Alkaline soil is widely distributed in China. Its rational utilization is an effective measure to solve land shortage and improve the environment. Alfalfa is characterized by strong salt and alkali tolerance and high yield and protein content. Nitrogen (N) and phosphorus (P) are the main nutrients for plant growth, and N metabolism is one of the primary forms of plant metabolism, which plays a vital role in quality and yield formation. The exploration of the effect of N and P on N metabolism and alfalfa growth will provide a theoretical basis for scientific fertilization for alfalfa in the alkaline soil of the Yinchuan Plain of the Hetao Basin. Therefore, a 2-year experiment of N and P addition was conducted. Six treatments were set up with a randomized block design, including without N (WN), medium N (MN), high N (HN), without P (WP), medium P (MP), and high P (HP). It was found that the MN and MP treatments increased plant height, stem diameter, stem/leaf, dry/fresh, and dry matter of alfalfa. The HN and HP treatments inhibited alfalfa biomass formation. The MN and MP treatments increased key products and enzymes of leaf N metabolism of alfalfa and promoted activities of leaf nitrate reductase (NR), glutamine synthase (GS), glutamate synthase (GOGAT), glutamic-oxalacetic transaminase (GOT), and glutamic-pyruvate transaminase (GPT), and inhibited activities of leaf protease of alfalfa. The MN and MP treatments increased contents of leaf N, P, ammonium nitrogen (NH_4_^+^-N), nitrate nitrogen (NO_3_^−^-N), total chlorophyll, and protein and reduced leaf chlorophyll a/b and amino acid, results after HN and HP treatments were opposite. The correlation among leaf P, N, NO_3_^−^-N, amino acid, and protein reached significant levels (*P* < 0.01). It is suggested that MN and MP treatments can improve the yield and quality of alfalfa by increasing key products and enzymes of N metabolism and can be adopted to promote alfalfa production in the alkaline soil of the Yinchuan Plain of the Hetao Basin.

## Introduction

Nitrogen (N) is an essential component of protein, nucleic acid, chlorophyll, coenzyme, and vitamin. It is one of the essential plant nutrients (Baslam et al., 2020) and an important factor for plant growth and crop yield (McAllister, Beatty & Good, 2012; Xu, Fan & Miller, 2012). The N metabolism is one of the most basic and dominant metabolisms, constituting the two most important metabolisms together with carbon metabolism in plants (Muro-Pastor & Hess, 2020). It is also a significant part of the geo-biological-chemical cycle. During N metabolism, plants synthesize inorganic N into organic N by nitrate reduction, ammonium nitrogen assimilation, amino acid synthesis, and protein (peptide chain) synthesis with the involvement of key enzymes and products. Nitrate reduction and ammonium nitrogen assimilation involve enzymes such as nitrate reductase (NR), glutamine synthase (GS), and glutamate synthase (GOGAT). Amino acid synthesis involves enzymes such as glutamic-oxalacetic transaminase (GOT) and glutamic-pyruvate transaminase (GPT), and protein synthesis involves enzymes such as peptidase and protease (Torre & Ávila, 2021; Guan et al., 2012). Several studies have shown that the intensity of N metabolism plays an important role in the growth, yield and quality of plants (Li et al., 2007; Wang, 2006).

Relevant studies have shown that N and phosphorus (P) are closely related to N metabolism in plants. The N acquisition, transportation, and assimilation are optimized under minimum or optimal N content (Maurya, Bandyopadhyay & Prasad, 2020). Wang (2017) found that N fertilizer of 180–270 kg/hm^2^ enhanced the activities of NR and GS and increased the yield of maize. Wang et al. (2021b) found that N sources in different forms also have different effects on metabolism, and NH_4_^+^-N contributes to amino acid accumulation and protein synthesis in tea plants. These results suggest that N fertilization has a positive effect on N assimilation, amino acid synthesis, and protein synthesis. The P is a main component of phospholipids in the skeleton of a biological membrane. It is essential for the normal division and metabolism of nucleic acid and nuclear protein of genetic material in plants. The effect of P on N metabolism in plants has not been studied as extensively as that of N. Naeem et al. (2010) found that P addition of 75 mg/kg increases chlorophyll content and carotenoid content and promotes photosynthesis, and increases the protein content by 14.9% in a pot experiment. Lu et al. (2020) found that the optimum foliar P concentration of peanut under sufficient P supply and P stress in soil was 0.1% and 0.2%, respectively, which promotes the activity of key enzymes of N metabolism in peanuts, such as NR, glutamate dehydrogenase (GDH), and GS. It has also been shown that the addition of P fertilizer can promote N accumulation in crops (Naeem et al., 2010; Chen et al., 2009).

Salinization causes many problems, such as land shortage, ecological deterioration, and soil fertility decrease (Jing et al., 2020) and is an important factor limiting the efficient use of regional ecosystems and the development of agriculture (Ondrasek & Rengel, 2021). The utilization of saline-alkali soil is a worldwide challenge (Talat, 2020). Saline-alkali soil is widely distributed in China, covering an area of around 99.13 million hm^2^, about 10% of the land area of China (Shang et al., 2019). Salinization is especially serious in the arid and semi-arid regions in northwest China, where the Yinchuan Plain of the Hetao Basin is the most important agricultural area and the most fragile ecological area (Wang, 2021). Due to the arid climate, intense evaporation, and unreasonable irrigation system (Zhang, 2020; Chu & Chen, 2018), the saline-alkali soil increased year by year in this area. In addition, serious degradation of soil quality and fertility has become the main obstacle to land utilization in the Yinchuan Plain of the Hetao Basin. The reasonable and effective use of saline-alkali land is significant to solve land scarcity, food insecurity and environmental pollution in the Yinchuan Plain of the Hetao Basin.

Relevant studies have presented plants that can adapt to saline and alkaline environment, especially plants with strong tolerance to salt-alkali soil, such as s*orghum bicolor ‘Dochna’*, *wolfberry, elaeagnus angustifolia, echinochloa frumentacea*, and alfalfa. Their salt-alkali tolerance can mainly be explained by physiological mechanisms, molecular mechanisms, and external phenotypes (Rani, Kumar & Suneja, 2021; Wang et al., 2021a; Zhao et al., 2020). Alfalfa is a high-yielding, high-quality, salt-tolerant, cold-resistant, and drought-resistant perennial leguminous herbage (Sun et al., 2020b). It increases the content of N, P, and soil organic carbon (SOC) and reduces the pH and alkalinity of soil (Li et al., 2019). In addition, alfalfa is a pioneer species for improving salt-alkali soil (Bhattarai et al. 2021), which is important for biological improvement, land use, and environmental protection. We speculate that the amounts of N and P may exert promoting or inhibiting effects on the key products and enzymes of N metabolism in the leaf of alfalfa, which directly affect the productivity of alfalfa in alkaline areas. At present, few single-factor N and P experiments have been conducted on alfalfa in alkaline soil, especially studies on the effects of N and P addition on the leaf N metabolism of alfalfa. In this study, two 2-year independent experiments of N and P were carried out for alfalfa in salt-alkali soil in the Yinchuan Plain of the Hetao Basin to analyze the effects of N and P on alfalfa performance and leaf N metabolism. This study provides theoretical support for the improvement of the yield and quality of alfalfa in alkaline soil.

## Materials and Methods

### Site description

**Figure 1:**
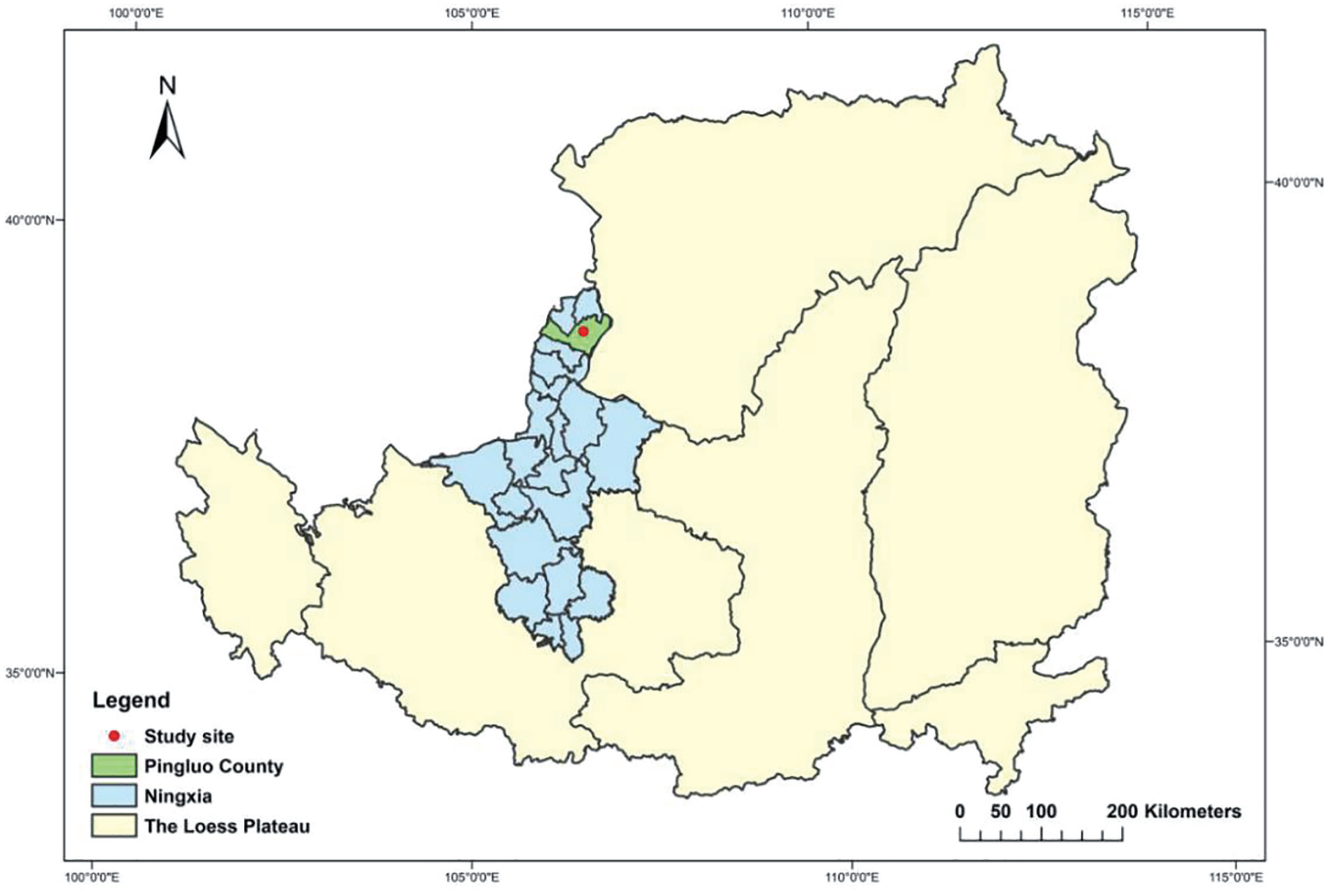
Location map and site of research.

The experiment was carried out in early July 2019 at the experimental base of Qianyeqing Agricultural Technology Development Co., Ltd, Pingluo County, Ningxia Hui Autonomous Region, China (38°50′N, 106°15′E; 1,091–1,110 m above sea level). The location map of the research site is shown in Fig. 1. Pingluo is a desert area, where solar radiation from April to October is 4,225.9 kJ/m^2^, and the strongest solar radiation occurs in June and is 716 kJ/m^2^. The annual cumulative sunshine hours range from 2,800 to 3,200, with the most in June. In addition, this area has little rainfall and much evaporation. The annual precipitation is 200–270 mm, and 66.6% of precipitation occurs from July to September. The maximum evaporation is 1,500–1,800 mm. The average annual humidity is about 56%. The average annual temperature is 6.8 °C, and the effective accumulated temperature above 10 °C is 1,638–2,638 °C. The frost-free period is about 150 d. The soil type of the test field is alkaline soil, and the salt ions mainly include Na^+^, Cl^−^, and SO_4_^2−^. The physical and chemical characteristics of the tested soil are shown in Table 1.

**Table 1:** Physical and chemical characteristics of the tested soil.

Index	SOC (g/kg)	OM (g/kg)	TN (g/kg)	NH_4_^+^-N (mg/kg)	NO_3_^−^-N (mg/kg)	TP (g/kg)	AP (mg/kg)	AK (mg/kg)	ESP (%)	pH
Depth										
20 cm	16.04	27.65	1.18	11.30	193.96	5.34	112.91	126.97	12.32	8.40
40 cm	10.52	18.14	0.97	7.41	256.21	3.92	42.06	130.99	14.47	8.50
60 cm	8.12	14.00	0.86	9.12	286.87	3.14	121.57	143.05	16.87	8.57

**Note:**

The SOC, OM, TN, NH_4_^+^-N, NO_3_^−^-N, TP, AP, TK, ESP, AK refer to organic carbon, organic matter, total nitrogen, ammonium nitrogen, nitrate nitrogen, total phosphorus, available phosphorus, alkalinity, available potassium, respectively.

### Experiment design and field management

**Figure 2:**
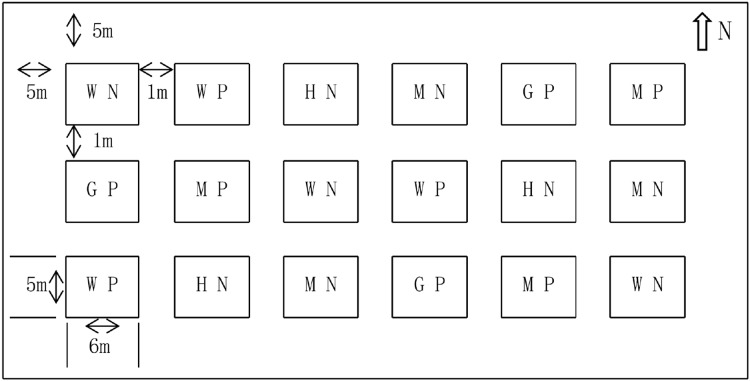
Field layout of nitrogen and phosphorus treatments.

**Figure 3:**
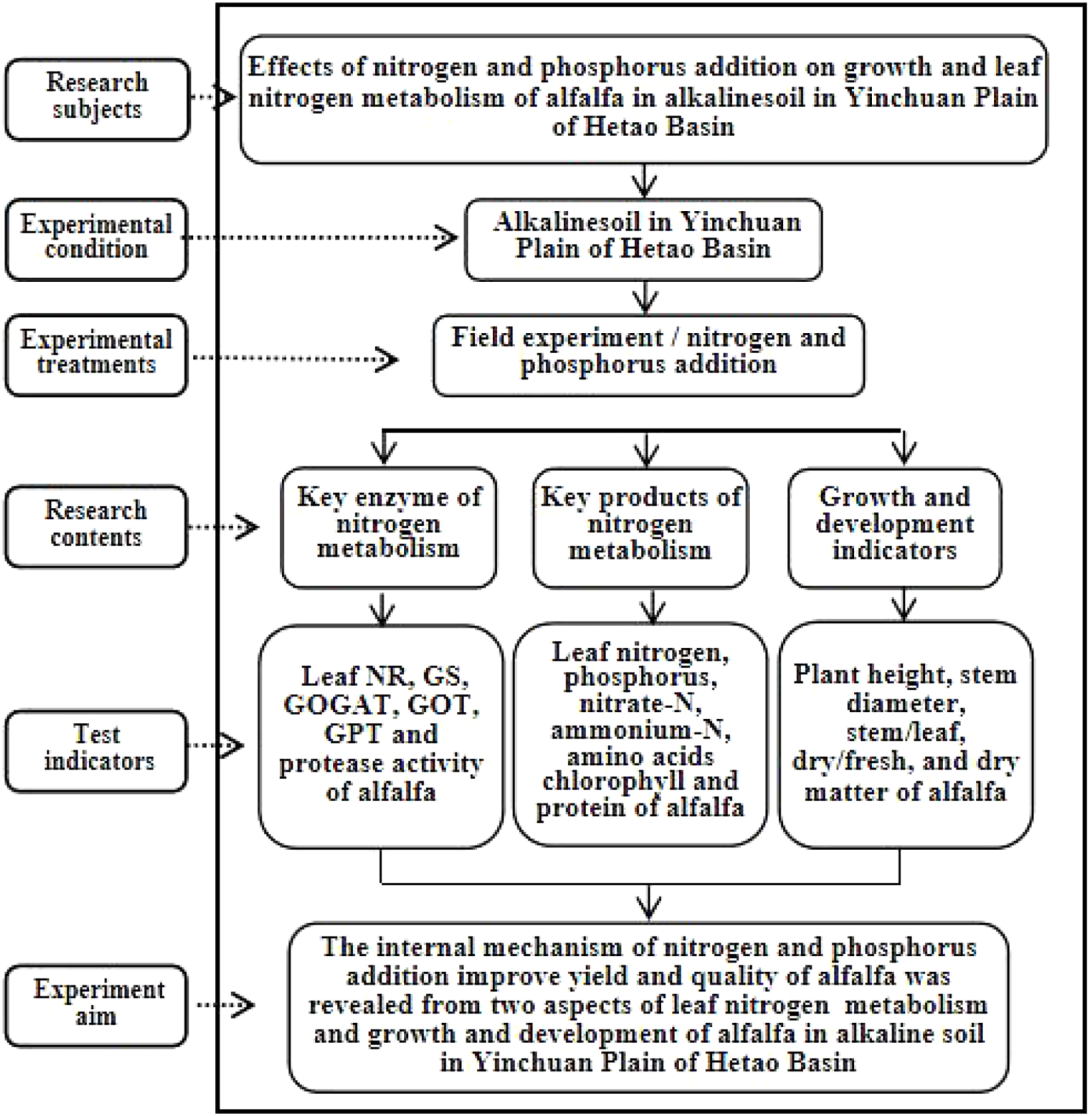
Technical roadmap of research.

The experiment adopted a randomized block design. Six treatments were set up, including without N (WN), medium N (MN), high N (HN), without P (W), medium P (MP), and high P (HP). The amount of N in WN, MN, and HN treatments were set as 0, 6, and 12 kg/667 m^2^, respectively. The P and potassium were used as supplementary for N treatment and their amount was 8 kg/667 m^2^ and 5 kg/667 m^2^, respectively. The amount of P in WP, MP, and HP treatments was set as 0, 9, and 18 kg/667 m^2^, respectively. The N and potassium were used as supplementary and their amount was 6 kg/667 m^2^ and 5 kg/667 m^2^, respectively. Each treatment was repeated three times and 18 blocks were obtained. The blocks were manually ridged using the ridge technique. The interval of the blocks was 1 m, the guard row of the test field was 5 m, the area of a single block was about 30 m^2^ (5 m × 6 m), the area of the experiment was 540 m^2^, and the actual area was 970 m^2^ (Fig. 2). In mid-April 2017, alfalfa was mechanically sown with a sowing quantity of 1.5 kg/667 m^2^ and a planting interval of 30 cm. Afterward, the alfalfa was harvested manually on June 10, July 15, August 20, and October 10, and after each harvest, a 5-cm stubble was left. In addition, irrigations to a depth of 5 cm were carried out on May 15, June 15, July 15, and August 15. The technical roadmap of the research is shown in Fig. 3. Fertilizer was manually added, with N added in half in April and half in July each year. The P was added once a year in April, and supporting fertilizer was added once a year in April. The variety of alfalfa was the “Salt-tolerant star” (Ningxia Qianyeqing Agricultural Technology Development Co., Ltd., Ningxia, China). Urea (N ≥ 46.4%) was used as N fertilizer, heavy calcium superphosphate (P_2_O_5_ ≥ 46%) was used as P fertilizer and potassium sulfate (K_2_O ≥ 50%) was used as potash fertilizer.

#### Sampling

Alfalfa in the same growth status was selected and labeled in the field every year, and the leaves on the top of the main branch of the labeled alfalfa were collected at the early flowering stage. With referring to the plant material preservation method of Hu (2017), the leaves were immediately frozen in liquid N and stored in the −80 °C refrigerator for the determination of leaf protein. Dry weight and fresh weight of selected alfalfa were determined by referring to the methods described by Pérez-Harguindeguy et al. (2013) and Garnier et al. (2010). The alfalfa was cut with a 5-cm stubble left and weighed. Then the alfalfa was dried at 105 °C for 30 min and dried to constant weight at 80 °C for the calculation of dry/fresh weight. Each treatment was repeated six times. Alfalfa yield was determined by referring to the method described by Guo et al. (2021). The 1 m^2^ sample method was used to determine the fresh weight of alfalfa, which was used to calculate the biomass of alfalfa in the field. Each treatment was repeated six times.

**Figure 4:**
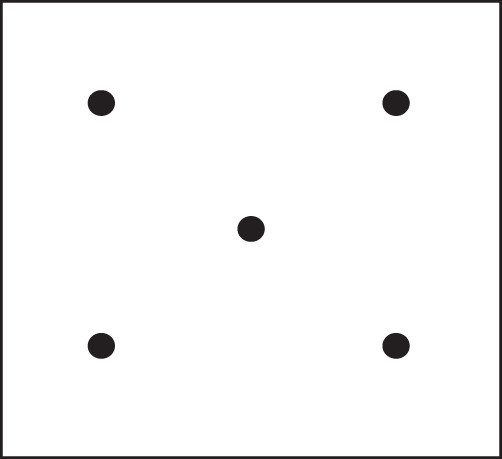
Sampling map of five-point method of field.

Non-rhizosphere soils of alfalfa were collected using the method described by Bao (2000). The five-point method (Fig. 4) was adopted to collect non-rhizosphere soil samples with a soil auger (approximately 5 cm in diameter). Three layers of soil were taken from the non-rhizosphere soil of alfalfa, with one layer being taken every 20 cm from the surface. The rhizosphere soil of alfalfa was collected by referring to the rhizosphere soil collection method for wheat described by Gqozo et al. (2020). Ten to fifteen plants of well-growing alfalfa were selected from each collection point. The soil attached to the root of alfalfa was collected as rhizosphere soil samples by shaking the root after the alfalfa was uprooted. The collected soil samples were air-dried, ground, and passed through 0.25, 1 and 2 mm sieves for laboratory analyses (The collection of plant samples and soil samples in the field has been approved by Qianyeqing Agricultural Technology Development Co., Ltd., and will not affect the future management of the field).

#### Determination

Determination of physicochemical characteristics of soil:

According to the American soil texture classification system, soil can be divided into sand (0.05–2 mm), silt (0.002–0.05 mm) and clay (<0.002 mm). Soil passed through the 1 mm mesh sieve was used to determine NH_4_^+^-N, NO_3_^−^-N, available P (AP), and available potassium (AK) contents. Soil passed through the 0.25 mm mesh screen was used to measure organic carbon (OC), organic matter (OM), total N (TN), total P (TP), and exchangeable sodium percentage (ESP) contents.

Soil OC, TN and AP contents were respectively determined using the Walkley-Black, Kjeldahl, and Olsen methods (Page, Miller & Kenney, 1982). Soil OM content was obtained by multiplying OC content by a conversion factor of 1.724. Soil AK content was quantified by 1 mol/L ammonium acetate extracts *via* flame photometry (PinAAciie 900F; PerkinElmer, Waltham, MA, USA). Soil TP content was determined using molybdenum antimony blue calorimetry (Murphy & Riley, 1962). Soil NH_4_^+^-N and NO_3_^−^-N were measured with a continuous flow analyzer (AutoAnalyzer-AA3; Seal Analytical, Norderstedt, Germany) after extraction with 2 mol/L KCl (Kachurina et al., 2000). Soil pH was measured using a pH meter (FE 20; Mettler-Toledo, Greifensee, Switzerland) at a ratio of 1:2.5 (soil/water) after shaking the equilibration for approximately 30 min (Dick, Cheng & Wang, 2000). Exchangeable Na^+^ was extracted using ammonium acetate, and its concentration was measured with the Prodigy-7 ICP-AES. Cation exchange capacity (CEC) values were determined using the BaCl_2_ and NH_4_OAc methods, and ESP was then calculated using the equation (ESP = (Na^+^/CEC) × 100) described by Seilsepour, Rashidi & Khabbaz (2009).

Method for the determination of biomass indicators:

Height and stem diameter of alfalfa were measured with a meter stick and vernier caliper, respectively. The dry/fresh of alfalfa was the ratio of dry weight to fresh weight of alfalfa. The stem/leaf of alfalfa was the ratio of stem weight to leaf weight of alfalfa. The dry matter was the product of fresh weight and dry/fresh of alfalfa.

Method for determination of nutrient indexes in leaf:

Alfalfa leaves were boiled using the H_2_SO_4_–H_2_O_2_ heating digestion method, and leaf N was determined by the distillation. Furthermore, leaf P was determined by the vanadium-molybdenum-yellow method of colorimetry (Bao, 2000).

Method for determination of indicators of N metabolism in leaf:

Under the ice bath condition, 0.100 g of fresh alfalfa leaves were taken for indicator determination and thoroughly ground with a mortar. The stock solution of tissue samples was extracted with the kit and added to the reagents in the kit. The activities of NR, GS, GOGAT, GOT, GPT, and protease were determined by an ultraviolet spectrophotometer. In addition, the contents of NO_3_^−^-N, NH_4_^+^-N, amino acids, and proteins were determined by an ultraviolet spectrophotometer. The determination of each indicator was repeated three times. All indicators were determined strictly in accordance with the operational requirements of the kits. The kits for all indicators were purchased from Comin Biotechnology Co. Ltd. (Suzhou, China). The chlorophyll in leaves was determined by a mixed acetone-ethanol-water method (Bao, 2000).

### Data processing

Mapping was performed using Excel 2019, and statistical analysis was performed using DPS v7.05 and origin2019b.

## Results

### Effects of N and P addition on growth and dry matter of alfalfa in alkaline soil

As shown in Table 2, the effect of N addition on alfalfa biomass is more significant than that of P addition. The MN treatment significantly increased plant height, stem diameter, dry/fresh, stem/leaf, and dry matter of alfalfa compared to the WN treatment (*P* < 0.05). Compared to the WN treatment, the HN treatment decreased plant height, stem diameter, dry/fresh, stem/leaf, and dry matter of alfalfa, with the difference in stem diameter and the dry matter reaching a significant level (*P* < 0.05). Compared to the WP treatment, the MP treatment significantly increased plant height, stem diameter, dry/fresh, stem/leaf, and dry matter of alfalfa, with differences being significant (*P* < 0.05). The effect of the HP treatment on plant height, stem diameter, dry/fresh, stem/leaf and dry matter of alfalfa are opposite to that of the MP treatment. The differences in plant height, stem diameter, and dry matter between the HP treatment and WP treatment reach significant levels (*P* < 0.05). These changes indicate that MN and MP treatments promoted alfalfa biomass formation, and HN and HP treatments inhibited alfalfa biomass formation. In addition, the effect of N addition on alfalfa biomass was more significant than that of P addition.

**Table 2:** Effects of nitrogen and phosphorus addition on plant height, stem diameter, dry/fresh, stem/leaf and dry matter of alfalfa in alkaline soil.

Treatment	2020					2021				
	Height (cm)	Stem diameter (mm)	Dry/fresh	Stem/leaf	Dry matter (kg/667m^2^/stubble)	Height (cm)	Stem diameter (mm)	Dry/fresh	Stem/leaf	Dry matter (kg/667m^2^/stubble)
N fertilizer										
WN	112.5 ± 4.28 a	3.51 ± 0.19a	0.22 ± 0.01a	1.51 ± 0.05a	271.8 ± 14.52a	114.3 ± 4.17a	3.54 ± 0.19a	0.22 ± 0.01a	1.52 ± 0.11a	272.3 ± 15.23a
MN	123.2 ± 4.15b	3.81 ± 0.21b	0.23 ± 0.01a	1.63 ± 0.09a	323.6 ± 18.34b	124.5 ± 3.46b	3.81 ± 0.20a	0.24 ± 0.02b	1.64 ± 0.04b	328.9 ± 16.34b
GN	108.7 ± 6.20a	3.24 ± 0.13a	0.21 ± 0.01a	1.43 ± 0.10a	259.2 ± 15.62a	109.2 ± 5.34a	3.28 ± 0.16b	0.20 ± 0.01a	1.40 ± 0.06ac	251.4 ± 12.78c
LSD0.05	*	**	NS	NS	**	**	NS	*	*	*
P fertilizer										
WP	109.1 ± 5.61a	3.27 ± 0.20a	0.21 ± 0.01a	1.41 ± 0.10a	267.9 ± 13.21a	110.6 ± 5.23a	3.31 ± 0.18a	0.21 ± 0.01a	1.38 ± 0.05a	273.2 ± 14.32a
MP	118.9 ± 1.33b	3.7 ± 0.17b	0.22 ± 0.02a	1.52 ± 0.11a	312.8 ± 15.45b	119.6 ± 4.58a	3.66 ± 0.17b	0.23 ± 0.01b	1.52 ± 0.08b	308.4 ± 15.82b
GP	104.6 ± 4.48a	3.1 ± 0.18a	0.20 ± 0.01a	1.29 ± 0.06a	253.7 ± 14.23b	101.5 ± 5.77b	3.01 ± 0.21c	0.2 ± 0.01a	1.29 ± 0.11ac	252.7 ± 12.76c
LSD0.05	* *	**	NS	NS	**	*	*	*	*	*

**Note:**

Different lowercase letters of same column of same fertilizer indicates significant difference at level of 0.05.2. NS indicates no significant difference, asterisks (* and **) indicates significant difference at level of 0.05 and 0.01, respectively.

### Effects of N and P addition on leaf P, N, NO_3_^−^-N, and NH_4_^+^-N of alfalfa in alkaline soil

**Figure 5:**
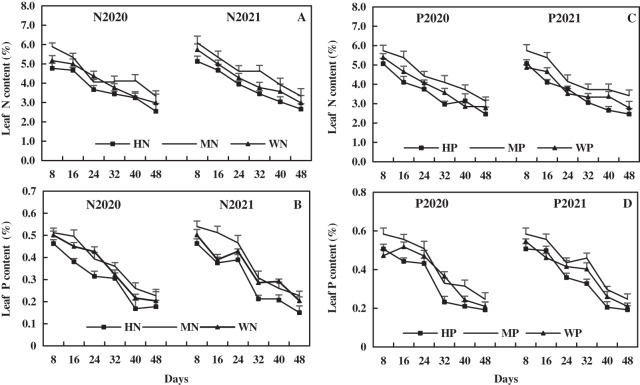
Effects of nitrogen and phosphorus addition on leaf nitrogen and phosphorus of alfalfa in alkaline soil.

The leaf N decreased with alfalfa growth, and the effect of N addition on leaf N of alfalfa was more higher than that of P addition. Compared to the WN treatment in 2020 and 2021, the MN treatment increased leaf N by an average of 6.09% and 10.16%, while the HN treatment decreased leaf N by 14.62% and 14.41%, respectively (Fig. 5A). Compared to the WP treatment, the MP treatment increased leaf N by 13.59% and 15.96% on average, and the HP treatment decreased leaf N by 7.81% and 6.29% in 2020 and 2021, respectively (Fig. 5C). These results suggest that MN and MP treatments increased the leaf N of alfalfa in alkaline soil, while HN and HP treatments had inhibitory effects.

Leaf P of alfalfa decreased with alfalfa growth, and the effect of P addition on leaf P of alfalfa was more higher than that of N addition. Compared to the WN treatment, the MN treatment increased leaf P content by 6.09% and 10.16% on average, but the HN treatment decreased leaf P content by 14.62% and 14.41% in 2020 and 2021, respectively (Fig. 5B). Compared to the WP treatment in 2020 and 2021, the MP treatment increased leaf P by 11.43% and 12.41% on average, but the HP treatment decreased leaf P by 11.53% and 8.98%, respectively (Fig. 5D). The results reveal that the MN and MP treatments increased leaf P of alfalfa in alkaline soil, while the HN and HP treatments had the opposite effect.

**Figure 6:**
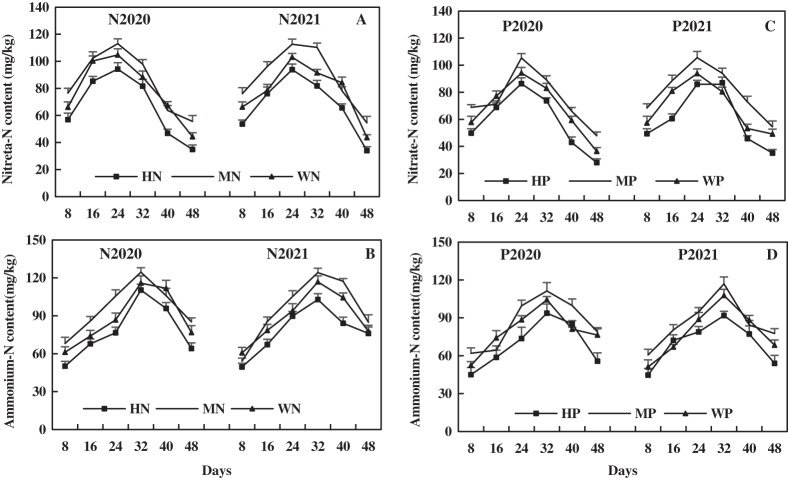
Effects of nitrogen and phosphorus addition on leaf nitrate-N and ammonium-N of alfalfa in alkaline soil.

The leaf NO_3_^−^-N of alfalfa increased first and then decreased with its growth, and the leaf NO_3_^−^-N after N addition was higher than that after P addition. In 2020 and 2021, compared to the WN treatment, the MN treatment increased the leaf NO_3_^−^-N by 8.12% and 13.04%, respectively, but the HN treatment decreased leaf NO_3_^−^-N by 15.11% and 13.27%, respectively (Fig. 6A). Compared to the WP treatment in 2020 and 2021, the MP treatment increased leaf NO_3_^−^-N by 10.01% and 16.70% on average, while HP treatment decreased leaf NO_3_^−^-N by 14.21% and 12.32%, respectively (Fig. 6C). The results indicate that MN and MP treatments increased the leaf NO_3_^−^-N of alfalfa in alkaline soil, and HN and HP treatments reduced leaf NO_3_^−^-N of alfalfa.

The leaf NH_4_^+^-N increased first and then decreased with alfalfa growth, and the effect of N addition on leaf NH_4_^+^-N of alfalfa was more higher than that of P addition. Compared to the WN treatment, the MN treatment increased the leaf NH_4_^+^-N of alfalfa by 9.29% and 7.38% on average, and the HN treatment decreased the leaf NH_4_^+^-N by 11.67% and 11.76% on average in 2020 and 2021, respectively (Fig. 6B). In addition, compared to the WP treatment in 2020 and 2021, the MP treatment increased the leaf NH_4_^+^-N of alfalfa by 8.22% and 9.28% on average, and the HP treatment decreased the leaf NH_4_^+^-N by 13.56% and 11.12% on average, respectively (Fig. 6D). The results indicate that MN and MP treatments increased leaf NH_4_^+^-N of alfalfa, while HN and HP treatments decreased the NH_4_^+^-N of alfalfa in alkaline soil.

**Figure 7:**
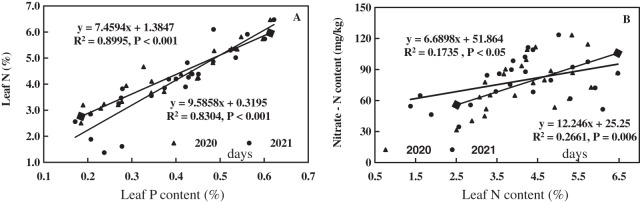
Effects of nitrogen and phosphorus addition on relationship of leaf phosphorus, nitrogen and nitrate-N of alfalfa in alkaline soil.

The linear equations of leaf P, N, and NO_3_^−^-N of alfalfa were fitted, as shown in Fig. 7. Leaf P of alfalfa was positively correlated with leaf N (R^2^ = 0.8304–0.8995**, *P* < 0.001**), and leaf N of alfalfa increased with increasing leaf P (Fig. 7A). Leaf N of alfalfa was positively correlated with leaf NO_3_^−^-N (R^2^ = 0.1735–0.2661**, 0.006** < *P* < 0.05**) and leaf NO_3_^−^-N of alfalfa also increased with the increase of leaf N (Fig. 7B). The results indicate a positive correlation between leaf N and P of alfalfa and between leaf N and NO_3_^−^-N in alkaline soil.

### Effects of N and P addition on leaf chlorophyll of alfalfa in alkaline soil

The N plays an important role in chlorophyll synthesis and is an essential component of chlorophyll, and therefore the amount of N can be reflected by chlorophyll content. It can be seen from Table 3 that the leaf chlorophyll increased first and then decreased with alfalfa growth. The leaf chlorophyll reached the peak at about day 32. The leaf chlorophyll after N addition was higher than that after P addition. The MN treatment significantly increased leaf chlorophyll of alfalfa (*P* < 0.05) and significantly decreased the leaf chlorophyll a/b in the middle and late periods of alfalfa growth compared to the WN treatment. Furthermore, the HN treatment decreased the leaf chlorophyll of alfalfa and increased the leaf chlorophyll a/b compared to the WN treatment. Compared to the WP treatment, the MP treatment increased leaf chlorophyll, with the difference reaching a significant level at day 32 (*P* < 0.05), and it also decreased leaf chlorophyll a/b. The HP treatment decreased the leaf chlorophyll and increased the leaf chlorophyll a/b of alfalfa compared to the WP treatment. The results suggest that MN and MP treatments promoted the formation of leaf chlorophyll and slowed the biosynthesis rate of leaf chlorophyll. Furthermore, the HN and HP treatments inhibited the formation of leaf chlorophyll, increased leaf chlorophyll a/b, and accelerated the decomposition of leaf chlorophyll b of alfalfa in alkaline soil.

**Table 3:** Effects of nitrogen and phosphorus addition on chlorophyll and chlorophyll a/b of alfalfa in alkaline soil.

Treatment	2020						2021					
	16 day		32 day		48 day		16 day		32 day		48 day	
	Chl a+b	Chl a/b	Chl a+b	Chl a/b	Chl a+b	Chl a/b	Chl a+b	Chl a/b	Chl a+b	Chl a/b	Chl a+b	Chl a/b
N fertilizer												
WN	36.3 ± 2.28a	3.1 ± 0.21a	57.4 ± 3.88a	2.9 ± 0.18ab	43.4 ± 2.12a	2.9 ± 0.19ab	35.2 ± 1.72a	2.9 ± 0.13a	55.2 ± 2.82a	2.9 ± 0.21ab	44.2 ± 1.98a	2.9 ± 0.17ab
MN	39.1 ± 0.67a	2.8 ± 0.16a	63.2 ± 2.72b	2.7 ± 0.15b	48.9 ± 2.03b	2.5 ± 0.17b	37.7 ± 1.40a	2.8 ± 0.17a	62.7 ± 3.17b	2.6 ± 0.15b	50.3 ± 2.74b	2.5 ± 0.19b
HN	35.2 ± 1.33a	2.9 ± 0.17a	54.1 ± 2.45ac	3.1 ± 0.20a	41.5 ± 1.82ac	3.1 ± 0.13a	33.4 ± 1.39a	3.1 ± 0.21a	51.1 ± 2.51ac	3.1 ± 0.22a	41.4 ± 2.22ac	3.2 ± 0.15a
LSD0.05	NS	NS	**	**	*	*	NS	NS	**	**	*	*
P fertilizer												
WN	34.5 ± 3.15a	2.7 ± 0.15a	55.1 ± 3.17a	2.8 ± 0.16ab	41.9 ± 1.74a	2.9 ± 0.17a	32.6 ± 1.62a	2.7 ± 0.16a	54.6 ± 2.05a	2.8 ± 0.18ab	42.9 ± 2.19a	2.9 ± 0.18a
MN	36.9 ± 0.52a	2.8 ± 0.17a	61.7 ± 2.95b	2.7 ± 0.13b	46.5 ± 1.75a	2.6 ± 0.16a	35.5 ± 1.52a	2.8 ± 0.17a	60.8 ± 2.46b	2.7 ± 0.17b	46.7 ± 1.85a	2.7 ± 0.16a
HN	33.1 ± 0.17a	2.8 ± 0.13a	52.2 ± 2.78ac	3.1 ± 0.19a	40.3 ± 2.23a	3.2 ± 0.19a	30.7 ± 1.27a	2.8 ± 0.19a	51.7 ± 2.74ac	3.0 ± 0.19a	40.8 ± 1.78a	3.1 ± 0.18a
LSD0.05	NS	NS	**	**	NS	NS	NS	NS	**	**	NS	NS

**Note:**

Different lowercase letters of same column of same fertilizer indicate significant difference at level of 0.05.2. NS indicates no significant difference, asterisks (* and **) indicate significant difference at level of 0.05 and 0.01, respectively.

### Effects of N and P addition on leaf amino acid and protein of alfalfa in alkaline soil

**Figure 8:**
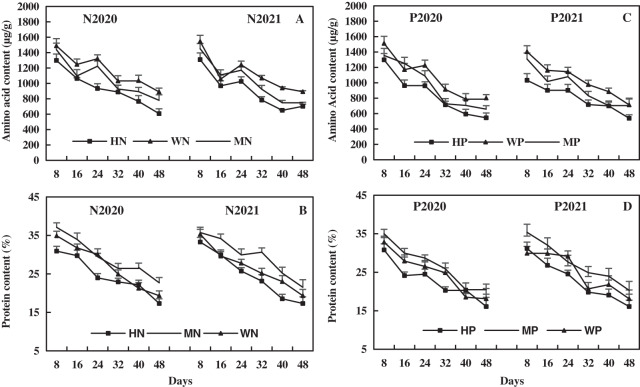
Effects of nitrogen and phosphorus addition on leaf amino acid and protein of alfalfa in alkaline soil.

The leaf amino acid decreased with alfalfa growth, and the effect of N addition on leaf amino acid was higher than that of P addition (Fig. 8). In 2020 and 2021, compared to the WN treatment, the MN treatment decreased leaf amino acid by 9.35% and 8.80% on average, and the HN treatment decreased leaf amino acid by 20.75% and 19.34% on average, respectively (Fig. 8A). In addition, the MP treatment decreased leaf amino acid by 9.78% and 10.20% on average and the HP treatment decreased leaf amino acid by 20.83% and 23.71% on average compared to the WP treatment in 2020 and 2021, respectively (Fig. 8C). The results reveal that the N and P addition decreased the leaf amino acids of alfalfa in alkaline soil, and the decrease became more significant with the increase of N and P addition.

The leaf protein decreased with alfalfa growth, and the effect of N addition on leaf protein of alfalfa was higher than that of P addition. Compared to the WN treatment, the MN treatment increased leaf protein by an average of 8.63% and 10.63% and the HN treatment decreased leaf protein by an average of 9.38% and 7.63% in 2020 and 2021, respectively (Fig. 8B). The MP treatment increased leaf protein by 7.80% and 9.85% on average, while the HP treatment decreased leaf protein by 8.44% and 8.03% on average compared to the WP treatment in 2020 and 2021, respectively (Fig. 8D). The results indicate that the MN and MP treatments increased alfalfa leaf protein, and HN and HP treatments had the opposite effect. The effect of N addition on alfalfa leaf protein was more significant than that of P addition.

**Figure 9:**
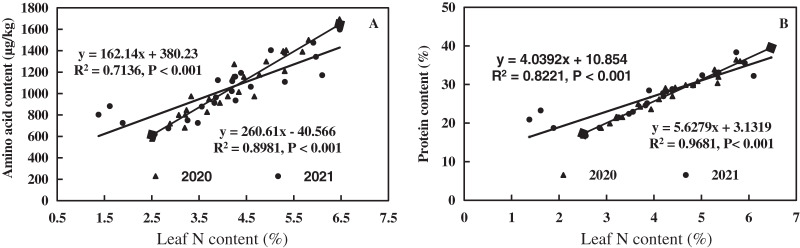
Effects of nitrogen and phosphorus addition on relationship of leaf nitrogen, amino acid and protein of alfalfa in alkaline soil.

The linear equations of leaf N, amino acid, and protein content of alfalfa were fitted, as shown in Fig. 9. Leaf N of alfalfa was positively correlated with leaf amino acid (0.7136 < R^2^ < 0.8981, *P* < 0.001), and leaf amino acid of alfalfa increased with the increase of leaf N (Fig. 9A). Leaf N of alfalfa was positively correlated with leaf protein (0.8221 < R^2^ < 0.9681, *P* < 0.001), and leaf protein of alfalfa also increased with the increase of leaf N (Fig. 9B). The results indicate a positive correlation between leaf N and amino acid and between leaf N and leaf protein of alfalfa in alkaline soil.

### Effects of N and P addition on key enzyme activity of N metabolism of alfalfa’s leaf in alkaline soil

**Figure 10:**
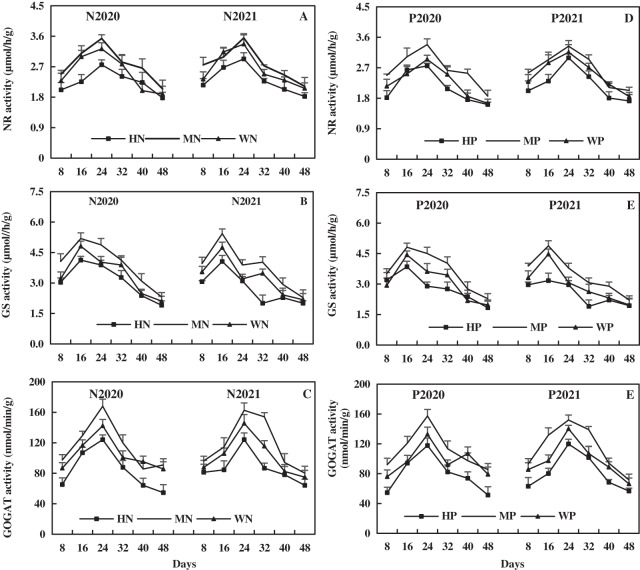
Effects of nitrogen and phosphorus addition on leaf activities of nitrate reductase (NR), glutamine synthase (GS) and glutamate synthase (GOGAT) of alfalfa in alkaline soil.

With the growth of alfalfa, leaf NR activity increased and then decreased, and leaf NR activity was relatively higher after N addition than after P addition. Compared to the WN treatment, the MN treatment increased leaf NR activity by 9.74% and 5.83% on average, while the HN treatment decreased leaf NR activity by 11.29% and 11.38% on average in 2020 and 2021, respectively (Fig. 10A). In addition, the MP treatment increased leaf NR activity by 16.72% and 5.65% on average, while the HP treatment decreased leaf NR activity by 7.07% and 11.84% on average compared to WP treatment in 2020 and 2021, respectively (Fig. 10D). The results indicate that MN and MP treatments increased leaf NR activity, while HN and HP treatment inhibited leaf NR activity of alfalfa in alkaline soil.

The leaf GS activity increased and then decreased with the growth of alfalfa, and the leaf GS activity after N addition was higher than that after P addition. The MN treatment increased the leaf GS activity by 15.34% and 15.05% on average, while the HN treatment decreased the leaf GS activity by 9.34% and 15.57% on average compared to WN treatment in 2020 and 2021, respectively (Fig. 10B). Moreover, compared to the WP treatment, the MP treatment increased the leaf GS activity by 17.88% and 16.78% on average, while the HP treatment decreased the leaf GS activity by 8.85% and 14.66% on average in 2020 and 2021, respectively (Fig. 10E). The results indicate that the MN and MP treatments increased the leaf GS activity of alfalfa in alkaline soil, while the HN and HP treatments had opposite effects.

The leaf GOGAT activity increased and then decreased with the growth of alfalfa, and the effect of N addition on leaf GOGAT activity was higher than that of P addition. Compared to the WN treatment, the MN treatment increased the leaf GOGAT activity by 10.24% and 14.69% on average, while the HN treatment decreased the leaf GOGAT activity by 19.80% and 15.21% on average in 2020 and 2021, respectively (Fig. 10C). In addition, the MP treatment increased the leaf GOGAT activity by 14.32% and 16.45% on average, while the HP treatment decreased the leaf GOGAT activity by 18.72% and 16.27% on average compared to the WP treatment in 2020 and 2021, respectively (Fig. 10F). The results reveal that the MN and MP treatments increased leaf GOGAT activity, while the HN and HP treatments inhibited leaf GOGAT activity of alfalfa in alkaline soil.

**Figure 11:**
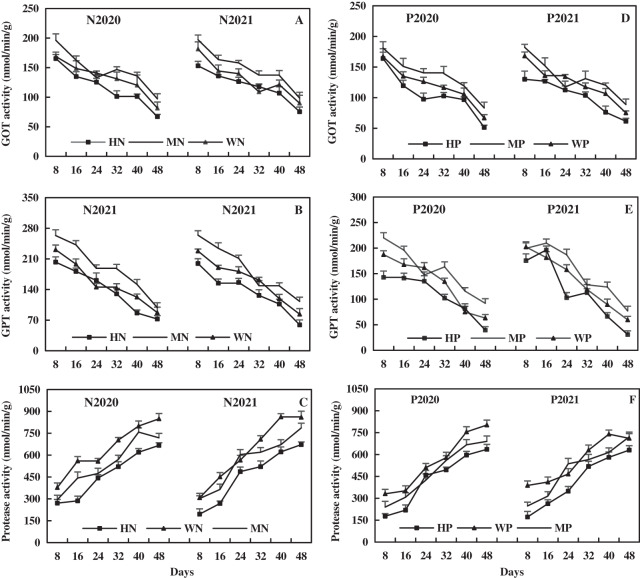
Effects of nitrogen and phosphorus addition on leaf activities of glutamic-oxalacetic transaminase (GOT), glutamic-pyruvate transaminase (GPT) and protease of alfalfa in alkaline soil.

The leaf GOT activity decreased rapidly first, then slowly, and then rapidly with the growth of alfalfa. The leaf GOT activity after N addition was relatively higher than that after P addition. Compared to the WN treatment, the MN treatment increased the leaf GOT activity by 10.21% and 13.48% on average, while the HN treatment decreased the leaf GOT activity by 12.07% and 8.89% in 2020 and 2021, respectively (Fig. 11A). In addition, the MP treatment increased the leaf GOT activity by 13.61% and 7.35% on average, while the HP treatment decreased the leaf GOT activity by 11.85% and 17.16%, compared to the WP treatment in 2020 and 2021, respectively (Fig. 11D). The results show that the MN and MP treatments increased leaf GOT activity of alfalfa in alkaline soil, while the HN and HP treatments caused opposite effects.

The leaf GPT activity decreased with alfalfa growth, and the effect of N addition on leaf GPT activity of alfalfa was relatively higher than that of P addition. In 2020 and 2021, the MN treatment increased the leaf GPT activity by 22.11% and 16.16% on average, while the HN treatment decreased the leaf GPT activity by 10.04% and 16.69% on average compared to the WN treatment (Fig. 11B). As shown in Fig. 11E, compared to the WP treatment, the MP treatment increased leaf GPT activity by 18.44% and 14.27% on average, while the HP treatment decreased leaf GPT activity by 18.59% and 15.44% on average in 2020 and 2021, respectively. These results indicate that the MN and MP treatments increased leaf GPT activity on alfalfa in alkaline soil, while the HN and HP treatments produced opposite effects.

As shown in Fig. 11, the leaf protease activity increased with the growth of alfalfa, and the effect of N addition on it was more higher than that of P addition. Compared to the WN treatment, the MN treatment decreased the leaf protease activity by 15.30% and 10.76% on average, while the HN treatment decreased the leaf protease activity by 27.01% and 26.30% on average in 2020 and 2021, respectively (Fig. 11C). Compared to WP treatment, the MP treatment decreased leaf protease activity by 13.22% and 10.35% on average, while the HP treatment decreased leaf protease activity by 22.46% and 24.90% on average in 2020 and 2021, respectively (Fig. 11F). The results reveal that N and P addition inhibited the leaf protease activity of alfalfa in alkaline soil, and the inhibitory effect became more significant with the increase of the addition.

## Discussion

### Effects of N and P addition on the growth, leaf nutrients, and chlorophyll of alfalfa in alkaline soil

The MN and MP treatments increased plant height, stem diameter, stem/leaf, dry/fresh, and dry matter of alfalfa in alkaline soil (*P* < 0.05), while HN and HP treatments exerted opposite effects (Table 2), suggesting that N and P have important on the growth and development of alfalfa. Many studies have shown that the application amount of N and P affects crop growth (Lemaire et al., 2021; Amanullah & Stewart, 2013; Zhang, 2014). It has been found that the low N amount of 352 g/plant can improve the yield and quality of bananas in red loam compared to the high N amount of 455 g/plant (Sun et al., 2020a). It has also been found that the yield and thousand-grain weight of wheat and maize intercropping increase firstly and then decrease with the increase of N and P, and the yield index of wheat is the best when N application rate is 191.7–216 kg/hm^2^ and P application rate is 165.38–186.64 kg/hm^2^. The yield index of maize is the best when the N application rate is 243–299.14 kg/hm^2^ and the P application rate is 168.38–189 kg/hm^2^ (Liu et al., 2014). These results strongly proved the finding of this study that MN and MP treatments can promote the productivity of alfalfa in alkaline soil while the HN and HP treatments exert opposite effects.

In this study, the MN and MP treatments increased leaf N, P, NO_3_^−^-N, and NH_4_^+^-N of alfalfa in alkaline soil, but HN and HP treatments exerted opposite effects (Figs. 5 and 6). The HN and HP treatments reduced leaf nutrient content, possibly because the increase of nutrient concentration inhibits alfalfa root activity and affects root nutrient absorption. Many studies have shown that N and P can promote nutrient absorption and accumulation as well as crop growth (Lemaire et al., 2021; Amanullah et al., 2013). A study has revealed that absorption of N and P by cotton increases first and then decreases with P addition, and the nutrient absorption of cotton reaches the maximum with P fertilizer of 75 kg/hm^2^ (Wei et al., 2018). Additionally, the absorption of N and P by pepper increases first and then decreases with N addition (He, 2016), which proves the results of this study. The increase of leaf N, P, NO_3_^−^-N, and NH_4_^+^-N after MN and MP treatments promote the absorption and assimilation of N, improving the efficiency of N cycling of alfalfa and promoting the growth and morphogenesis of alfalfa. The effect of HN and HP treatment on alfalfa is the opposite.

Chlorophyll is an important pigment involved in the photosynthesis of plants. It exists in the thylakoids in the form of the pigment protein complex and plays an important role in capturing and transmitting light energy during photosynthesis (Logan, 2007). Chlorophyll exists in most photosynthetic organisms with mere differences in amount and type and significantly affects the yield and quality of plants. In this study, the MN and MP treatments increased the total volume of leaf chlorophyll and decreased the chlorophyll a/b of alfalfa, while HN and HP treatments decreased the total leaf chlorophyll and increased the chlorophyll a/b of alfalfa (Table 3). Many studies have shown that chlorophyll a, chlorophyll b, and total leaf chlorophyll content of crop firstly increased and then decreased with the increase of N and P addition (Wang & Cao, 2010; Hou et al., 2021), which is consistent with the result of the present study. Chlorophyll a/b decrease first and then increase with the increase of N and P, which may be related to the alkaline soil, alfalfa varieties, and degradation or synthesis rate of chlorophyll b under HN and HP treatments. It was found that leaf chlorophyll a/b of tea (Baojing Gold Tea No.1) decreased first and then increased with the increase of N addition, and in pot experiments with N addition of 2.28 g/kg, 1.52 g/kg, 0.76 g/kg and 0 g/kg, chlorophyll a/b was the lowest with N addition of 1.52 g/kg (Xiang et al., 2018), which proves the results in the present research. It was also found that leaf chlorophyll a/b of corn with N addition of 150 kg/hm^2^ was the lowest (Wu et al., 2019). It can be concluded that MN and MP treatments can effectively allocate leaf chlorophyll a and chlorophyll b of alfalfa leaf and promote the photosynthesis of alfalfa, which will improve N metabolism of alfalfa leaf and the productivity of alfalfa in alkaline soil.

### Effects of N and P addition on leaf N assimilation and cycling of alfalfa in alkaline soil

Fertilization promotes the physiological and biochemical processes of crops (Wang, Li & Malhi, 2008; Sperling et al., 2019). The N increases the activity of acid invertase (AI), sucrose synthase (SS), and sucrose phosphatase (SPS) (Yu et al., 2017). The P can improve the biochemical properties of asparagus (Cocozza et al., 2020). Similarly, N and P have certain effects on the N metabolism of crops. Zhang (2014) found that the N application rate of 750 kg/hm^2^ could improve the activity of NR, GS, GOGAT, and content of soluble protein, free amino acid and chlorophyll in functional leaves of cucumber. Ruiz & Romero (2000) found a positive effect of high P fertilization dose (8 and 16 g/m^2^) on the uptake, translocation and assimilation of NO_3_^−^-N in the leaves compared to the low P dose (4 g/m^2^), which proves the results of this study. It can be concluded that N and P could increase the leaf N metabolism of alfalfa, which is conducive to the yield formation and quality of alfalfa in alkaline soil. There is a significant positive correlation among leaf P, N, NO_3_^−^-N, amino acid, and protein (Figs. 7 and 9), suggesting that N and P improve the yield and quality of alfalfa mainly through enhancing the metabolic intensity of leaf N metabolism pathway of alfalfa in alkaline soil. Similar results were found in the leaf of dwarf bamboo and poplar (Shi, 2018; Liu et al., 2015). Therefore, the N and P have a promoting effect on the N metabolism of plants.

The NR, GS, and GOGAT are important catalysts and regulators for reduction and assimilation of NO_3_^−^-N and NH_4_^+^-N in plants. The NO_3_^−^-N is the initial product of the assimilation of most N-containing compounds. The NR is the key enzyme of NO_3_^−^-N assimilation and is the enzyme of the first step that catalyzes and regulates the absorption of NO_3_^−^-N (Casiraghi et al., 2020). The GS and GOGAT together form the GS/GOGAT cycle and regulate the assimilation of NH_4_^+^-N. The GS is an enzyme that catalyzes the synthesis of glutamine from NH_4_^+^-N and glutamate. The GOGAT catalyzes the transformation from the amino of glutamine to α-ketoglutaric acid and the formation of two-molecule glutamates. In this study, the MN and MP treatments increased the activity of NR, GS, and GOGAT and increased the NO_3_^−^-N and NH_4_^+^-N contents in the leaf of alfalfa in alkaline soil (Figs. 6 and 10), while HN and HP treatments brought about opposite results, indicating that MN and MP treatments had positive effects on N metabolism. It was found that P application rate of 126 kg/hm^2^ could significantly promote the activity of NR and GS and improve the efficiency of N metabolism in alfalfa compared to 0 kg/hm^2^ and 252 kg/hm^2^ (Yu, Liu & Hao, 2018). Wang et al. (2018) found that N Limitation (7.5 mM) can promote the activity of leaf NR, nitrite reductase (NiR), and GS of achnatherum compared to without N treatment. These results indicated that the MN and MP treatments enhance the NR, GS and GOGAT activity of N metabolism in crops, promote the absorption and assimilation of N, and improve the efficiency of N metabolism. It can be concluded that the MN and MP treatments can promote the absorption, reduction, and assimilation of N in the leaf of alfalfa and improve the yield formation and quality of alfalfa in alkaline soil.

Glutamate is the most basic amino acid and the most important amino acid in NO_3_^−^-N assimilation. It can be synthesized into other amino acids or proteins under catalysis and regulation of various enzymes. The GOT and GPT are key enzymes that promote the transformation of glutamic acid into other amino acids, and their level can reflect the level of amino acid metabolism (Suzuki, 2021). In this study, the MN and MP treatments increased the activities of leaf GOT and GPT of alfalfa in alkaline soil and decreased the contents of amino acids. In contrast, the HN and HP treatments decreased leaf activities of GOT and GPT of alfalfa in alkaline soil and also decreased the contents of amino acids (Figs. 8 and 11). Theoretically, the MN and MP treatments will increase the leaf amino acid of alfalfa. However, this study achieved an opposite result, possibly because MN and MP treatments rapidly promote the conversion of amino acids into proteins or may be similar to the result that deficiency of potassium significantly inhibits the transport of amino acids from leaf source to leaf sink (Wang et al., 2012). In addition, a study found that the composition and content of amino acid of the seedling of *eucalyptus globulus* are inversely related to the amount of P addition (Wang, 2009), which also supports the results of the present study to a certain extent. In the present study, the MN and MP treatments promoted leaf GOT and GPT activity of alfalfa in alkaline soil and accelerated the synthesis of amino acids in alfalfa.

Protease is a group of enzymes in living organisms that breaks down protein. The strength or weak of protease activity can reflect the amount of protein, and the decomposition of protease breaks peptide bonds that bind amino acids into polypeptide chains (Lisak Jakopović et al., 2019). It was found that N and P fertilizers increase the crude protein and total protein of alfalfa and cucumber (Yu, Liu & Hao, 2018; Zhang, 2014). In this study, all N and P treatments reduced leaf protease activity of alfalfa, and the inhibitory effect of HN and HP treatment was more significant, reducing the degradation of leaf protein. The MN and MP treatments increased leaf protein of alfalfa (Figs. 8 and 11). Wang et al. (2003) found that the protease activity of flag leaves decreases with the increase of N application rate, and the protease activity is the lowest at the N application rate of 240 kg/hm^2^ in wheat. Moreover, Cai, Ge & Zu (2007) found that a P application rate of 0.033–0.067 g/kg can increase the leaf soluble protein of different soybean genotypes. The P can promote the assimilation of NH_4_^+^-N and NO_3_^−^-N of soybean and enhance respiration, and various organic acids formed by respiration can act as ammonia receptors to generate amino acids and increase protein content (Cai, Ge & Zu, 2007). In the present study, the HN and HP treatments reduced leaf protein of alfalfa (Fig. 8), which might be related to the alkaline soil and alfalfa varieties. It can be concluded that the MN and MP treatments decrease leaf protease activity and increase the protein content of alfalfa, which is beneficial to the accumulation of protein content and the production of high-quality alfalfa.

All in all, the MN and MP treatments could improve the yield and quality of alfalfa mainly through the promotion of the leaf N metabolism of alfalfa in alkaline soil in the following three steps. Firstly, MN and MP treatments improve the assimilation of NO_3_^−^-N and NH_4_^+^-N through increasing activities of NR, GS and GOGAT. Secondly, MN and MP treatments increase the content of amino acids by improving the activity of GOT and GPT. Finally, MN and MP treatments increase the content of protein by decreasing the activity of protease and degradation of protein. The HN and HP treatments decrease the yield and quality of alfalfa by inhibiting the leaf N metabolism of alfalfa in alkaline soil.

## Conclusions

First, the MN and MP treatments improved plant height, stem diameter, stem/leaf, dry/fresh, and dry matter of alfalfa in alkaline soil. The HN and HP treatments had opposite effects.

Second, the MN and MP treatments improved the activities of leaf NR, GS, GOGAT, GOT, and GPT but decreased the activities of leaf protease in alkaline soil. The effect exerted by HN and HP treatments was the opposite.

Third, the MN and MP treatments improved contents of leaf N, P, NO_3_^−^-N, NH_4_^+^-N, total chlorophyll, and protein, but decreased leaf chlorophyll a/b and amino acids content of alfalfa in alkaline soil. The HN and HP treatments had an inhibitory effect.

In summary, the MN and MP treatments promoted leaf N metabolism of alfalfa and improved yield and quality.

## Supplemental Information

10.7717/peerj.13261/supp-1 Supplemental Information 1 Raw data.

10.7717/peerj.13261/supp-2 Supplemental Information 2 Report on paper repetition rate.

## Additional Information and Declarations

### Data Availability

The following information was supplied regarding data availability: The raw data is available in the Supplemental File.
