# Supplementary material for: Effects of nitrogen and phosphorus addition on growth and leaf nitrogen metabolism of alfalfa in alkaline soil in Yinchuan Plain of Hetao Basin
Source: PeerJ. 2022 Apr 13;10:e13261. doi: 10.7717/peerj.13261 (PMC9013234; doi:10.7717/peerj.13261)
Supplement: Supplemental Information 2 [file peerj-10-13261-s002.pdf]

PaperWord检测报告-打印版

检测文献：Main manuscript2（无参考文献）（免费版）
文献作者：
报告时间：2021-11-22 15:01:56
段落个数：5
报告编号：W202111221501318448
检测范围： 中国期刊库 中国图书库 硕士论文库 博士论文库 会议论文库 报纸库 网友专利库 网友标准库 网友共享库 个人对比库 网页库 百科库

总文字复制比：6.1%

去除引用文献复制比：6.1%　　去除本人已发表文献复制比：6.1%

单篇最大文字复制比：0.7%

重复字数：2,174	总字数：35,699（不含参考文献）
总段落数：5（不含参考文献）	前部重合字数：117
疑似段落数：5	后部重合字数：2,057
单篇最大重复字数：264	疑似段落最小重合字数：96

1.Main manuscript2（无参考文献）_第1部分　　**总字数：6,890**

文字复制比：3.8%（262）

1	Research<br>- 《网页 》-	1.2%<br>是否引证：否
2	硅氮添加对高寒草甸优势物种披针叶黄华生长及净光合速率的影响<br>徐当会;李秋霞;张仁懿 - 《草业科学 》- 2020	1.2%<br>是否引证：否
3	М Е Ж Д У Н А Р<br>- 《网页 》-	1.2%<br>是否引证：否
4	N、P停止施入后植物叶片主要元素含量及化学计量特征的响应<br>韩潇潇;林力涛;于占源;刚群;黄月;林贵刚;曾德慧 - 《生态学杂志 》- 2020	1.2%<br>是否引证：否

2.Main manuscript2（无参考文献）_第2部分 总字数：7,060

文字复制比：12.7%（900）

1	Combined application of a straw layer and flue gas desulphurization gypsum to reduce soil salinity and alkalinity<br>YongganZHAO;YanLI;ShujuanWANG;JingWANG;LizhenXU - 《土壤圈:英文版 》- 2020	3.9%<br>是否引证：否
2	生态沟渠吸收氮磷效果研究<br>杨勇;田昌;谢桂先;张玉平;宋海星;荣湘民 - 《农业科学与技术:英文版 》- 2019	1.9%<br>是否引证：否
3	Int.J.En<br>- 《网页 》-	1.3%<br>是否引证：否
4	PlantSoi<br>- 《网页 》-	1.2%<br>是否引证：否
5	Professi<br>- 《网页 》-	1.1%<br>是否引证：否
6	2006Frui<br>- 《网页 》-	1.1%<br>是否引证：否
7	AJCS7(8)<br>- 《网页 》-	1%<br>是否引证：否
8	SolidEar<br>- 《网页 》-	1%<br>是否引证：否
9	J.Agric.<br>- 《网页 》-	1%<br>是否引证：否
10	7thSASTe<br>- 《网页 》-	1%<br>是否引证：否

3.Main manuscript2（无参考文献）_第3部分 总字数：7,405

文字复制比：1.3%（96）

1	Seediscu<br>- 《网页 》-	1%<br>是否引证：否
2	Developmental Threshold Accumulated Temperature Cresson Temperature and Effective of Lysiphlebus testaceipes<br>Sun Chengpeng[1,2];Li Gangtie[1];Liu Aiping[2] - 《Plant Diseases and Pests(植物病虫害研究：英文版）》- 2017	1%<br>是否引证：否
3	2011Prev	1%

- 《网页 》-	是否引证：否

4.Main manuscript2（无参考文献）_第4部分　　总字数：7,904

文字复制比：8%（635）

1	收稿日期：201<br>- 《网页 》-	1.1%<br>是否引证：否
2	Internat<br>- 《网页 》-	1%<br>是否引证：否
3	西藏地区白草幼苗期抗旱性比较研究（英文）<br>张光雨;王江伟;张豪睿;付刚;沈振西 - 《Journal of Resources and Ecology 》- 2020	1%<br>是否引证：否
4	潮汐式生物接触氧化短程硝化反硝化强化脱氮研究<br>- 《网页 》-	1%<br>是否引证：否
5	河套灌区小麦套种玉米高产水氮高效利用生理机制<br>- 《网页 》-	0.9%<br>是否引证：否
6	河套灌区春玉米—土壤系统对不同水氮运筹模式的响应及DSSAT-CERES-Maize模型的适用性研究_农业博士论文_笔耕文化传播<br>- 《网页 》-	0.9%<br>是否引证：否
7	黑土微生物活力对不同养分的响应 - 生态环境学报<br>- 《网页 》-	0.9%<br>是否引证：否

5.Main manuscript2（无参考文献）_第5部分　　总字数：6,439

文字复制比：4.4%（281）

1	A time-splitting pressure-correction projection method for complete two-fluid modeling of a local scour hole<br>KambizFarahiMoghadam;MohammadAliBanihashemi;PeymanBadiei;AliShirkavand - 《国际泥沙研究:英文版 》- 2020	2.9%<br>是否引证：否
2	Bark Residues Recovery of Juglans Regia. L For the Dyeing of Wool Fabrics : Development of Microwave-assisted Extraction and Dyeing Processes: Journal of Natural Fibers: Vol 0, No 0<br>- 《网页 》-	2.3%<br>是否引证：否
3	电化学剥离石墨烯作为高性能催化剂载体促进甲醇在铂催化剂上的电催化氧化（英文）<br>袁旭;岳文博;张锦 - 《Journal of Central South University 》- 2020	1.9%<br>是否引证：否
4	640Cazet<br>- 《网页 》-	1.2%<br>是否引证：否

5	FACULTEI<br>- 《网页 》-	1.1%<br>是否引证：否

说明：

1.由于篇幅原因，本打印报告单最多只展示最相关的10条相似源

2.总文字复制比：被检测论文总重合数在总字数中所占的比例

3.去除引用文献复制比：去除系统识别为引用的文献后，计算出来的重合数字在总数字中所占比例

4.去除作者本人已发表文献后，计算出来的重合字数在总字数中所占的比例

5.单篇最大文字复制比：被检测文献与所有相似文献对比后，重合字数占总字数的比例最大的那一边文献的文字复制比

6.指标是由系统根据《学术论文不端行为的界定标准》自动生成的

7.本报告单仅对您所选择比对资源范围内检测结果负责

版权所有 www.paperword.com

扫码查看报告
